# Isoniazid-induced gynaecomastia: report of a paediatric case and review of literature

**DOI:** 10.1186/s12902-020-00639-9

**Published:** 2020-10-27

**Authors:** Sarah Wing Yiu Poon, Ka Ka Siu, Anita Man Ching Tsang

**Affiliations:** Department of Paediatrics and Adolescent Medicine, The University of Hong Kong, Queen Mary Hospital, Pok Fu Lam, Hong Kong

**Keywords:** Gynaecomastia, Isoniazid, Paediatric

## Abstract

**Background:**

Gynaecomastia is a fairly common condition in puberty but is rare in prepubertal boys. While it is necessary to exclude possible endocrinopathay in prepubertal gynaecomastia, medication is an important and potentially reversible cause to consider in new onset gynaecomastia. Isoniazid-induced gynaecomastia has been reported in adult males, but none was reported in the paediatric population and general paediatricians may not be aware of this uncommon side effect.

**Case presentation:**

We hereby report a 11-year-old prepubertal boy who developed gynaecomastia while taking anti-tuberculosis drugs. Investigations excluded endocrinopathies. Gynaecomastia subsided 8 weeks after stopping isoniazid.

**Conclusion:**

This case is the first paediatric case report describing the association of gynaecomastia with isoniazid use. It is important for general paediatricians to recognize this entity, as prompt diagnosis and cessation of the offending drug can lead to resolution of the problem.

## Background

Isoniazid has selective bactericidal activity for mycobacteria [1]. It is a key component of anti-tuberculosis therapy and is well tolerated by children and adolescents with low rates of serious adverse effects. Common side effects include rash, fever, hepatitis, peripheral neuropathy and gastrointestintal upset. Gynaecomastia is a rare adverse effect. There were ten case reports of isoniazid-induced gynaecomastia in adults, while none was reported in children. Here we describe a case of gynaecomastia in an 11-year-old prepubertal boy after receiving therapeutic dose of isoniazid for 4 months. This case highlights the importance of recognizing the temporal association of this rare side effect of isoniazid during the course of anti-tuberculosis therapy.

## Case presentation

An 11-year-old boy, who has good past health and normal development, presented with 4-day history of fever, cough and haemoptysis. Appetite was maintained and he did not have any chills or rigors. There was no sick contact. Examination showed body weight 26.3 kg (10th centile) and height 140.9 cm (55th centile) according to the local growth chart [2]. There was decreased air-entry over right middle and lower zone on chest examination. Investigations showed normal white cell count 13.8 × 10^9/L but elevated erythrocyte sedimentation rate (ESR) 29 mm/hr. (ref < 12 mm/hr) and lactate dehydrogenase (LDH) 1078u/L (ref 380-750u/L). Mantoux test was positive with 10 mm induration. Chest x ray showed volume loss over the right lung field with consolidation over right middle and lower zone with tracheal deviation to the right. He was initially treated with augmentin but consolidation persisted. Bronchoscopy was then performed which showed suspicious stenosis at the right middle lobe bronchus. Smear for acid-fast bacilli and polymerase chain reaction (PCR) for *Mycobacterium tuberculosis* (MTB) of sputum, early morning gastric aspirate, early morning urine (EMU) and bronchoalveolar lavage (BAL) were all negative. The child was initially treated as latent tuberculosis and started on isoniazid 250 mg daily (10 mg/kg). In view of the abnormal bronchoscopy findings, computerised tomography (CT) thorax was arranged which revealed segmental collapse of the apical segment of right lower lobe with matted lymph nodes. In view of the CT changes, he was treated as active pulmonary tuberculosis. Rifampicin 400 mg daily, Pyrazinamide 800 mg daily and Ethambutol 400 mg daily, were added in addition to isoniazid. Subsequent cultures of sputum, gastric aspirate, EMU and BAL for MTB were negative.

Four months after isoniazid treatment (i.e. 3 months after full anti-tuberculosis treatment), he complained of painful left breast swelling. Clinical examination showed a 1.5 cm × 1 cm firm glandular tissue around the nipple-areola complex on the left side and a 0.5 × 0.5 cm glandular tissue on the right side. Both swellings were mildly tender on palpation. There were no skin changes or nipple retraction. There were no palpable axillary lymph nodes. Abdominal examination was normal. He did not have significant weight gain during that period and his body weight was 27.5 kg (10th centile). He was pre-pubertal with Tanner stage 1 genitalia, testes 3 ml in volume and of normal consistency and no pubic hair development. There was no history of exposure to any other drugs, exogenous forms of estrogens or topical use of essential oils. There was no family history of gynaecomastia. Laboratory investigations showed normal renal, liver and thyroid functions; prepubertal levels of leutinizing hormone < 0.1 IU/L (< 1.0–12 IU/L), follicle stimulating hormone 0.36 IU/L (1.0–12 IU/L) and testosterone 0.2 nmol/L (adult range 10–35 nmol/L). Estradiol was slightly elevated at 30 pmol/L (adult range 20–160 pmol/L, prepubertal < 20 pmol/L). Prolactin level was normal at 229 mIU/L (45–375 mIU/L). Tumour markers including alpha fetoprotein and beta-human chorionic gonadotropin (b-HCG) were unremarkable. Ultrasound breasts showed bilateral small discs of retroareolar fibroglandular tissue compatible with gynaecomastia. With the above normal investigation results, we suspected isoniazid was the culprit. Isoniazid was then withheld and rifampicin, pyrazinamide and ethambutol were continued for a total of 6 months. Breast tissue subsided 8 weeks after withholding isoniazid.

## Discussion and conclusion

Gynaecomastia is defined as presence of palpable breast tissue in males and histologically characterised by benign glandular proliferation of breast tissue. Clinically it appears as a rubbery or firm, concentric mass at the areolar-nipple complex, in contrast to “pseudogynaecomastia” or fatty breast that occurs frequently in obese men, which is soft in consistency due to fat deposition without glandular proliferation. Gynaecomastia is common particularly in the newborn period, at puberty, and in the elderly. It occurs in up to 70% of all boys at pubertal period [3]. It may cause psychological distress due to change in body image or even painfulness.

Gynaecomastia primarily results from an imbalance of androgenic and estrogenic influence on breast tissue. Estrogen stimulates the proliferation of breast tissue whereas androgen inhibits it. Gynaecomastia occurs when there is an increase in circulating or tissue level of estrogen, decrease in circulating or tissue level of androgen, altered serum androgen/estrogen ratio, increased breast tissue sensitivity to estrogen (e.g. due to increased number of estrogen receptors) or decreased breast tissue sensitivity to androgen (e.g. androgen receptor defect or drugs). Common causes of gynaecomastia include obesity, ageing, primary or secondary hypogonadism, liver or renal failure, hyperthyroidism and less commonly feminizing adrenal or testicular tumour (e.g. Leydig or Sertoli cell tumour), gonadal or extragonadal HCG producing tumours and genetic conditions resulting in aromatase excess or androgen insensitivity [3].

In contrast to pubertal gynaecomastia which is common, prepubertal gynaecomastia is rare. Medical evaluation is warranted to rule out endocrinopathies, estrogen or HCG producing tumours or aromatase excess. History of exposure to lavendar oil or tea tree oils should be sought as the components in these essential oils had been demonstrated in vitro to have estrogenic and anti-androgenic effects and their use had been reported to cause prepubertal gynaecomastia [4].

Medication is a reversible cause of gynaecomastia and a careful drug history has to be taken. In healthy young males, particular attention should be made to use of anabolic steroid in body-builders and recreational use of marijuana. Drugs can cause gynaecomastia by its estrogenic activity (e.g. digitoxin), by decreasing serum testosterone (e.g. ketoconazole, metronidazole by damaging or inhibiting Leydig cells), blocking androgen receptor (e.g. spironolactone, cimetidine, marijuana), increasing serum prolactin (e.g. antipsychotics, metoclopramide) or via unknown mechanisms (e.g. antidepressants, human growth hormone, highly active antiretroviral therapy, proton pump inhibitors) [3]. Among the drugs in the standard tuberculosis regimen, only isoniazid has been reported to be associated with gynaecomastia.

In our patient, clinical examination and laboratory studies showed no evidence of liver, renal or thyroid disorder. Tumour markers were also taken to rule out the possibility of a HCG-producing tumour which may cause stimulation of Leydig cells. He was not obese and did not have excessive weight gain during the course of treatment, making obesity and refeeding gynaecomastia less likely. His gonadotrophins were within the pre-pubertal range while estradial was mildly elevated for his age and sex.

Isoniazid is known to induce pyridoxine (vitamin B6) deficiency by inhibitng pyridoxal phosphokinase and by combining with pyridoxine to form isonicotinylhydrazide which is excreted in urine [5]. Apart from causing the well-known side effect of peripheral neuropathy, isoniazid was also postulated to cause altered estrogen-androgen metabolism. The physiologically active form of vitamin B6, pyridoxal 5-phosphate (PLP), acts as a modulator of steroid hormone receptor- mediated gene expression. Elevation of intracellular PLP leads to a decreased transcriptional response to glucocorticoid hormones, progesterone, androgens, and estrogens, while cells in a vitamin B6-deficient state exhibit enhanced responsiveness to steroid hormone [6]. This might thus account for the elevated estradial level in our patient. Another possible mechanism is “refeeding gynaecomastia” associated with weight gain during anti-tuberculosis therapy, but this was not seen in our case.

We performed a literature search in Pubmed and Medline using text terms “isoniazid” and “gyneacomastia”. Animal studies and articles written in a language other than English were excluded. Ten articles describing such phenomenon in adult patients were found (Table 1) [7–16]. Age of presentation ranges from 18 to 72 years old, with mean age of 40.9 years old. Gynaecomastia occurred 3–6 months (mean 4.5 months) after isoniazid treatment, and subsided at variable time frame ranging from 1 month to 1 year. Bilateral breasts were involved in 60% (*n* = 6) of the cases. Our reported case is the youngest among all and is the only reported pre-pubertal case. Like most of the reported cases, there was bilateral involvement of the breasts. The clinical course of our patient resembles the reported adult cases, with gynaecomastia occurring 4 months after isoniazid intake. Breast enlargement resolved 8 weeks upon cessation of medication in our case. The relatively quick resolution might be related to the absence of pre-existing breast tissue in pre-pubertal males.

**Table 1:** Case reports of isoniazid-induced gynecomastia in the English literature

Author (yr of publication)	Age (in yr)	Diagnosis	Treatment	Onset of gynaecomastia after ATT	Clinical features	Outcome
1. Khanna et al. (2003) [7]	25	Right TB pleural effusion	2HRZE/2HR, H 300 mg, R 450 mg, Z 1500 mg, E 800 mg	4 m	Right painful breast 5x4cm Left painless nipple swelling	Bilateral breast swellings persisted for 3 m after ATT stopped, but decreased slightly and non-tender; subsided at 1 yr
2. Dixit et al. (2008) [8]	42	TB cervical lymph node	2H_3_R_3_Z_3_/2H_3_R_3_/2REC, H 600 mg, R 450 mg, Z 1500 mg	4 m	Left painful breast 6x8cm	H stopped at 4th m and breast swelling subsided after 6 months
3. Morrone et al. (2008) [9]	18	Cavitatory pulmonary TB (2 episodes)	1st episode: 2HRZ/4HR 2nd episode: 2HRZ/4HR/6R, H 400 mg, R 600 mg, Z 2000 mg	1st episode: 3 m; 2nd episode: 6 m	1st episode: mildly tender small breast swellings 2nd episode: Right tender breast swelling 5 cm	Gynaecomastia remained 2 months after H stopped and resolved 2 m after 1 yr of ATT
4. Garg et al. (2009) [10]	60	Pulmonary TB	2HRZE/3HRE/4RE, H 300 mg, R 450 mg, Z 1000 mg, E 800 mg	5 m	Bilateral tender breast swellings 5x6cm	Breast swellings became non-tender after H stopped and resolved 6 m after 9 m of ATT
5. Mansoor et al. (2009) [11]	50	TB epididymo-orchitis	2HRZE/4HR, H 300 mg, R 450 mg, Z 1500 mg, E 800 mg	4 m	Bilateral painful breast swellings	After H stopped, breast swellings resolved at 6 m of ATT
6. Lee et al. (2009) [12]	72	Pulmonary TB	2HRZ_3_E_3_/2 HRE_3_/5 RE_3,_ H 300 mg, R 600 mg, Z 1000 mg, E 400 mg	4 m	Bilateral painful breast swellings 4 cm	Breast swellings resolved 1.5 m after H stopped
7. Agrawal et al. (2011) [13]	28	Pulmonary TB	2HRZE/4HR, H: 300 mg, R: 450 mg, Z: 1500 mg, E: 800 mg	3 m	Bilateral tender breast swellings 3x4cm	Breast pain and swellings subsided 2 m after H stopped
8. Goud et al.(2012) [14]	45	Pulmonary TB	2HRZE/2HR/5RE, H 300 mg, R 450 mg, Z 1500 mg, E 800 mg	4 m	Left painful breast swelling 5x4cm	Breast swelling resolved 1 m after H stopped
9. Khan (2012) [15]	45	Bilateral TB cervical lymph node	2H_3_R_3_Z_3_/1H_3_R_3_/3R_3_E_3,_ H 600 mg, R 600 mg, Z 1500 mg	3 m	Left painful breast swelling 4 cm	Pain subsided 1 m after H stopped and breast swelling decreased by 25% at 3 m after H stopped
10. Neki (2016) [16]	24	Pulmonary TB	2HRZE/2.5HRE/4.5RE, H: 300 mg, R: 450 mg, Z: 1500 mg, E: 800 mg	4.5 m	Bilateral tender breast swellings 4x3cm	Breast pain and swelling subsided 1 m after H stopped

*ATT* anti-tuberculosis therapy

Drugs, *H* Isoniazid, *R* Rifampicin, *Z* Pyrazinamide, *E* Ethambutol, *C* Cirpofloxacin

Duration: Shown by the number (in months) in front of the drugs, separated by the slash “/” to indicate another phase of treatment

Frequency: Shown by the subscript attached to the individual drug (i.e. subscript “3” indicates thrice weekly administration and no subscript indicates daily administration)

Most patients with drug induced gynaecomastia do not require treatment other than discontinuation of the offending agent. Likewise, no treatment was initiated in all the reported cases and our case. Reassurance is especially important in adolescents, and follow up should be arranged to document regression of breast tissue after the drug is withdrawn. Medical therapy, for example, antiestrogen and aromatase inhibitors are only indicated in those with significant pain or in those with considerable social anxiety due to breast enlargement.

This case is the first paediatric case report describing the association of gynaecomastia with isoniazid use. It is important for general paediatricians to recognize this entity, as prompt diagnosis and cessation of the offending drug can lead to resolution of the problem.

## Data Availability

The datasets used during the current study are available from the corresponding author on reasonable request.

## Abbreviations

ESR: Erythrocyte sedimentation rate
LDH: Lactate dehydrogenase
PCR: Polymerase chain reaction
MTB: *Mycobacterium tuberculosis*
EMU: Early morning urine
BAL: Bronchoalveolar lavage
CT: Computerised tomography
PLP: Pyridoxal 5-phosphate

## References

[1] Timmins GS, Deretic V (2006). Mechanisms of action of isoniazid. Mol Microbiol.

[2] Leung SSF, Tse LY, Wong GWK (1995). Standards for the anthropometric assessment of nutritional status of Hong Kong children. HKJ Paediatr.

[3] Narula HS, Carlson HE (2014). Gynaecomastia—pathophysiology, diagnosis and treatment. Nat Rev Endocrinol.

[4] Henley DV, Lipson N, Korach KS, Bloch CA (2007). Prepubertal gynecomastia linked to lavender and tea tree oils. N Engl J Med.

[5] Mandel W (1959). Pyridoxine and the isoniazid-induced neuropathy. Dis Chest.

[6] Oka T (2001). Modulation of gene expression by vitamin B6. Nutr Res Rev.

[7] Khanna P, Panjabi C, Maurya V, Shah A (2003). Isoniazid associated, painful, bilateral gynaecomastia. Ind J Chest Dis Allied Sci.

[8] Dixit R, Sharma S, Nawal CL (2008). Gynaecomastia during antituberculosis chemotherapy with isoniazid. J Assoc Physicians India.

[9] Morrone N, Morrone N, Junior BAG, Maia JA (2008). Gynecomastia: a rare adverse effect of isoniazid. J Bras Pneumol.

[10] Garg R, Vaibha V, Mehra S, Prasad R (2009). Isoniazid induced gynaecomastia: A case report. Indian J Tuberc.

[11] Mansoor T, Rizvi SA, Khurram F, Ali W. Isoniazid-Induced Gynaecomastia. Internet J Surg. 2009. p. 18. [Available from: http://www.ispub.com/journal/the-internet-journal-of-surgery/volume-18-number-1/isoniazid-induced-gynaecomastia.html].

[12] Lee MK, Jib Na D, Jeon H, Lee YD, Cho YS, Han MS (2009). A case of isoniazid induced Gynecomastia. Tuberc Respir Dis.

[13] Agrawal P, Gupta AK, Goyal V, Handa A, Gupta A (2011). Isoniazid-induced gynaecomastia. J Indian Acad Clin Med.

[14] Goud BKM, Devi OS, Nayal B, Devaki RN (2012). A rare case of unilateral gynecomastia during antituberculous chemotherapy with isoniazid. Indian J Pharmacol.

[15] Khan A, Agarwal R (2012). Isoniazid related gynecomastia: description of a case and systematic review of literature. Lung India Off Organ Indian Chest Soc.

[16] Neki NS (2016). Isoniazid-induced gynaecomastia. J Indian Acad Clin Med.

